# Lymphatic network in atherosclerosis: the underestimated path

**DOI:** 10.4155/fso.15.61

**Published:** 2015-11-01

**Authors:** Andreea Milasan, Jonathan Ledoux, Catherine Martel

**Affiliations:** 1 Montreal Heart Institute, Research Center, Montreal, QC, Canada; 2 Department of Medicine, Faculty of Medicine, Université de Montréal, Montreal, QC, Canada

**Keywords:** atherosclerosis, lymphatic endothelium, lymphatic smooth muscle cells, lymphatic vessels

## Abstract

The lymphatic system is a key component of tissue fluid homeostasis. In contrast to the closed and high-pressure blood vascular system, the lymphatic vascular system transports lymph in an open and low-pressure network. A prerequisite player in the transport of immune cells and cholesterol metabolism, it has been understudied until recently. Whereas defects in lymph circulation are mostly associated with pathologies such as congenital or acquired lymphedema, emerging significant developments are unraveling the role of lymphatic vessels in other pathological settings. In the last decade, discoveries of underlying genes responsible for developmental and postnatal lymphatic growth, combined with state-of-the-art lymphatic function imaging and quantification techniques, have matched the growing interest in understanding the role of the lymphatic system in atherosclerosis. With a historical perspective, this review highlights the current knowledge regarding interaction between the lymphatic vascular tree and atherosclerosis, with an emphasis on the physiological mechanisms of this multifaceted system throughout disease onset and progression.

## Abstract (lay abstract)

The blood and lymphatic vascular systems are parallel but interdependent networks. The lymphatic system governs the transport of superfluous interstitial fluids from peripheral tissues to the blood circulation, maintaining fluid balance throughout the body. Defects in lymphatic function have been broadly associated with pathologies such as congenital or acquired lymphedema. Although longstanding observations suggested that the lymphatic vasculature could be central in the development of chronic inflammatory diseases, recent publications specifically point out its potential implication in atherosclerosis. In this review, we highlight the current knowledge unraveling the interaction between the lymphatic network and atherosclerosis, with an emphasis on the physiological mechanisms of this intricate system.

In the 1620s, two important circuits of the bodies were officially discovered: *De Moto Cordis* and *De lactibus sive lacteis venis* were two independently published manuscripts, portraying the blood and the lymphatic circulation, respectively. Albeit interdependent, the blood and the lymphatic vascular networks are two separate circulatory systems running in juxtaposition, having separate, but often interdependent, functions [1]. Four centuries after their first official identification, advances on blood vascular biology far surpass those of lymphatic biology. Why has there been such a gap in the interest given to both particular systems? Whereas tools to study the venous and arterial circulation have been developed at a greater pace, exploring the lymphatic network had its load of difficulties. Until very recently, very few methods had been optimized to fully characterize lymphatic function in health and disease. The urge for a better understanding of this complex system became obvious with the report of the ubiquitous presence of those milky-white vessels in nearly all vascularized tissues. Their potential association with diverse severe pathologies was then clearer. The progress that has been made since the turn of the present century is tremendous: new genetic mouse models [2,3] and imaging tools [4,5] developed for both animals and humans have greatly contributed to unraveling the role of the lymphatic system in different pathophysiologic conditions even outside of the fields of lymphedema, such as in atherosclerosis.

This article highlights recent advances in our understanding of the role of the lymphatic vascular tree in cardiovascular diseases, with a specific attention to atherosclerosis. We place particular emphasis on work depicting the functional mechanisms of this complex system throughout disease onset and progression.

## Historical perspective

In Italy, 23 July 1622, a professor of anatomy and surgery was meticulously practicing vivisection on a dog. Gasparo Aselli and a group of colleagues were observing recurrent nerves in the animal. Subsequently, in an attempt to watch the movements of the diaphragm in the same operation, he opened the abdomen. Thinking he was dealing with nerves belonging to the intestines, the Italian described what he saw as ‘(…) cords, exceedingly thin and beautifully white, scattered the whole mesentery and the intestine, starting from innumerable beginnings.’ [6]. Therefore, in the first published color-printed illustrations in a medical or anatomical work, Professor Aselli depicted the ‘vessels containing white blood’ described by Hippocrates in 400 BC [7] as *lacteae venae*, or ‘milky veins’ [6]. Three decades later, Thomas Bartholin created the term ‘lymphatics,’ based on his work performed in parallel to the work of others on the thoracic duct in the same period (Olaus Rudbeck, 1630–1702 ; Jean Pecquet, 1624–1674 ; George Joyliffe, 1621–1658) [8]. While discoveries of the 17th century led to the establishment of the anatomical atlas of the lymphatic system, the discoveries from the two following century portrayed the network as a passive draining network, bringing a first insight on the role of the lymphatic system. William and John Hunter pioneered the discovery of the absorptive capacity of the lymphatics in the 18th century [9], while studies on lymph motion through the lymphatic vessels had started to emerge in the 19th century. Lymph composition *per se* was studied concomitantly, first suggesting that lymph is formed as a filtrate of the blood [10]. It is in the second end of that century that Rudolf Virchow investigated the role of lymph nodes as filtering units [11]. Few decades later, in the early 20th century, Florence Rena Sabin brought up the first insight on lymphatic development. She innovatively proposed lymph sacs originate from endothelial cells that outgrow from the veins during early development [12]. We had to wait for a couple of decades before observing lymphatics through lymphography, a method allowing the clinical observation of lymphatic disorders [13].

## General anatomy & functions of the lymphatic vessels

The lymphatic system is now recognized as working in close collaboration with the cardiovascular system. The lymphatic network is an essential component of the immune system, playing major roles in host defense and adaptive immunity, as it is the principal route of transport from tissues for antigen and immune cells [14]. Lymphatic vessels are required for the maintenance of fluid homeostasis within the body [15], absorbing lymph through thin-walled and blind-ended lymphatic capillaries (also called initial lymphatics) from the peripheral tissue. Initial lymphatics are highly permeable and constituted of specialized, discontinuous ‘button-like’ junctions between endothelial cells [16]. Lymphatic capillaries are characterized by an absence of smooth muscle cells (SMCs) and specific expression of LYVE-1 on the lymphatic endothelial cells (LECs). When lymph continues its path from the initial lymphatics, it converges into larger precollecting and subsequently collecting lymphatic vessels [15,17]. Collecting vessels are responsible of maintaining lymph flow through contraction of units called lymphangions. A lymphangion is defined as a vessel segment delimited by two endothelial leaflet valves. In the absence of strong positive pressure leading the lymph toward the vein, these valves allow unidirectional flow in preventing lymph backflow [16]. A basement membrane, podoplanin expression, continuous ‘zipper-like’ cell–cell junctions and a SMC layer are also distinguishing collecting vessels from lymphatic capillaries. After reaching the lymph nodes (LNs), the afferent collecting vessels become the efferent collecting vessels and, ultimately, the collecting lymphatic vessels converge into the thoracic duct or the right lymphatic trunk, where lymph is finally reaching the venous circulation via the subclavian vein [13,14]. In the intestines, the lymphatic system is essential as it absorbs lipid through entities called lacteals, transporting lipids from the gut into the blood [18,19].

Following up on Sabin’s work [12], it has been described that venous endothelial cells (VECs) differentiation into LEC progenitors from the cardinal vein allows lateral sprouting and lymph sacs formation, the latter being characterized by the expression of the transcription factor Prox1 [20]. Prox1^+^ cells require a paracrine VEGF-C gradient to spread away from embryonic veins [21]. Subsequently, the lymph sacs undergo extensive remodeling to form a structured vascular network. Lymphatic development and regulation are dependent upon VEGF-C/D and its receptor VEGFR-3 [22], and Prox1 drives VEGFR-3 expression that enables LEC to respond to VEGFR-3 ligands [23].

To properly determine whether lymphatics are active players in cardiovascular disease progression or regression, we must combine morphological observations and lymph composition with functional studies. Several parameters need to be taken into consideration when assessing proper lymphatic function, whether it is at a cellular or molecular level, both under physiological and pathological conditions.

## Lymphatic morphology in atherosclerosis

Lymphatic biology and function in heart disease is gaining exponential attention. First studied in the fields of lymphedema and cancer, the lymphatic vascular tree is now broadly studied in acute and chronic inflammatory diseases. Atherosclerosis is a chronic inflammatory disease affecting large- to medium-sized arteries, and driven by two main constituents: macrophages and cholesterol. Macrophages accumulate in the expanding aortic intima, engulf lipids (becoming so-called foam cells) and produce a wide-ranging spectrum of inflammatory mediators [24–27]. Macrophage reverse cholesterol transport (mRCT) is the mechanism by which cholesterol homeostasis occurs in the artery wall, as cholesterol is mobilized from foam cells and subsequently transferred from the peripheral tissue to the liver and feces [28], or directly to the fecal pathway [29]. Both exit of foam cells and cholesterol from the plaques are believed to be essential targets in the reduction of atherosclerosis burden, and risk of coronary events such as plaque rupture [30].

Surprisingly, despite the well-defined roles of lymphatic vessels in preserving fluid balance throughout the body by returning plasma proteins from interstitial spaces back to the blood circulation, only little attention has been given yet to the role of the lymphatic vasculature in the atherosclerotic process. In the last century, only a few avant-garde scientists pointed out the concept that lymphatics, and particulary lymph flow, could influence atherogenesis [31–33]. In a review manuscript published in 1981, Lemole had concatenated important publications in which intimal thickening was observed following lymphatic blockade. He suggested that enhanced stagnation of the interstitial fluid in the arterial wall could be due to lymphostasis, phenomenon in which factors that might contribute to the development of atherosclerosis, such as intimal edema, are present [33]. He concluded by suggesting that further studies in immunology, pathology, lipid metabolism and nuclear medicine were needed to confirm this hypothesis [33]. Thirty years later, several original fundamental studies and clinical observations have been published in this regard.

Interest to lymphatic biology and function in coronary artery disease is growing exponentially. Morphological analysis of the lymphatic vessels within the arterial wall gave the first insight of the association between the lymphatic system and atherosclerosis. In animal models, lymphatic vessels have been consistently observed in the adventitial and periadventitial regions of the artery wall [34,35]. Xu *et al.* suggested that the arrangement of the lymphatic vessels within the artery wall reflects the importance of this complex network in maintaining the drainage of local inflammatory cells and cytokines from peripheral tissue such as the adventitia [36]. In addition of being studied in dog epicardial coronary arteries, rabbit carotid and thoracic aorta, and rat aortic wall after balloon-induced aortic endothelial injury, the presence of lymphatic vessels have also been observed in hypercholesterolemic mice. In apoE^-/-^ fed on a high-fat western diet, lymphatic capillaries have been found in the adventitial layer, waving in and out of the adjacent adipose tissue of the aorta, and to be quite numerous beneath plaques in the aortic sinus [37].

In a clinical setting, Drozdz *et al.* took interest in the presence of lymphatics in the adventitia of the internal carotid artery in humans and showed that the number of adventitial lymphatics increases with severity of atherosclerosis measured as intimal thickness [38]. Where it becomes particularly interesting, is that they stipulate that arteries that are covered by a dense network of lymphatic vessels seem naturally protected against atherosclerosis when compared with those that lack such a network [39]. In addition, Kholova *et al.* have published that lymphatic vessels can also be found in the intima of advanced lesions [40]. On the other hand, other teams observed no [41] or very little [42] lymphatic vessels in the wall of normal or atherosclerotic human epicardial coronary arteries, despite the accentuated presence of VEGF-C [42].

Controversial morphological characterizations described above certainly deserve critical consideration. For example, what does the presence of lymphatic vessels in the adventitia of the aortic wall really means? Are plaque-associated lymphatic vessels friends or foes in atherosclerosis? Research groups are actively seeking for the answers, especially through altering lymphatic function in animal models of atherosclerosis.

## Lymphatic function in atherosclerosis

Three decades after Lemole reported observational studies considering a connection between intimal edema and lymphatics [33], genetic and surgical interventions in animal models have emerged, in the faith of getting better insights on the role of the lymphatic network in atherosclerosis.

Recently, a functional and quantitative study has reported the prerequisite role of the lymphatic system in the removal of cholesterol from the artery wall, putting front stage the importance of the lymphatic network in mRCT [37]. Using a surgical model of aortic transplant from a hypercholesterolemic apolipoprotein E-deficient (Apoe^-/-^) donor to a hypercholesterolemic Apoe^-/-^ receiver in which ApoE vector was injected to induce cholesterol efflux, it has been shown that the pattern of the newly regrown lymphatic vessels posttransplant is influenced by the blood flow through the transplant [37]. The lymphatic vessels thus formed appeared to be atheroprotective: in conditions where lymphatic vessels had fully grown posttransplant, the cholesterol contained in the transplanted artery was able to exit the atherosclerotic lesion. By contrast, partial inhibition of lymphatic regrowth using VEGFR-3 antibody was reflected by retention of cholesterol in the artery wall of the transplanted aortic segment [37].

Genetic manipulations in mice have also brought new insights on the link between impaired lymphatic vessels and atherogenesis. Primary congenital lymphedema (Milroy disease) is a rare autosomal dominant condition caused by mutations in the *vegfr-3* gene [3]. Primary human lymphedema is characterized by a chronic and disfiguring swelling of the extremities. A mouse model to study the physiological regulation of lymph flow and to assess the therapeutic potential of VEGF-C to stimulate lymphatic revascularization has been put forth by Karkkainen *et al.* [3]. Called ‘Chy mice’ because of their apparent development of chylous ascites after birth, this mouse model of lymphedema has an inactivating *vegfr-3* mutation in their germ line, causing a selective incomplete development of lymphatic vessels dermally and thus swelling of the limbs [2]. Furthermore, despite the complete loss of initial lymphatics in the limbs and ears resulting in impaired cell trafficking through lymph, the scarce lymphatics present in the body trunk reflected a normal dendritic cell transport [43]. An additional mouse model has been reported to inhibit the formation of the dermal lymphatic vasculature [44]. Mice expressing soluble VEGFR-3 under the keratin-14 (K14) promoter (K14-Vegfr-3-Ig) display a neutralized activity of VEGF-C and VEGF-D in the dermal lymphatic vasculature when expressed in mouse epidermis. They, therefore, also show impairment in transport of solutes and dendritic cells from the skin to the corresponding draining LNs [44]. Vuorio *et al.* took advantage of these characterized mouse models and analyzed the effects of the absence of lymphatics on lipoprotein metabolism and atherosclerosis [45]. Crossing each of these two transgenic mouse models baring lymphatic insufficiency with atherosclerotic mice (Ldlr^-/-^/Apob^100/100^), the group observed a positive correlation between atheroma formation and the absence of lymphatic vessels. They showed that both sVegfr-3×Ldlr^-/-^/Apob^100/100^ and Chy×Ldlr^-/-^/Apob^100/100^ mice have increased cholesterol levels leading to accelerated atherogenesis, suggesting that lymphatic vessels have an important role in maintaining proper lipoprotein metabolism and vascular homeostasis [45].

Inflammation being a critical part of the atherosclerotic process, studies of VEGF-C in inflammatory bowel disease (IBD) might be relevant to atherosclerosis. Crohn’s disease and several types of intestinal ulcers are diseases associated with an aberrant mucosal immune response. Recently, D’Alessio *et al.* showed remarkable results, which demonstrated that adenoviral induction of prolymphangiogenic factor VEGF-C provides marked protection against the development of acute and chronic colitis in two different animal models [46]. They explained the protective function of VEGF-C as being meditated by the ‘resolving macrophages,’ as they call them, in a STAT6-dependent manner. This VEGF-C/VEGFR-3 pathway that seems to regulate macrophage plasticity and activation proves hopeful for the correction of defective lymphatic function especially when it comes to the process of plaque formation in atherosclerosis, particularly in mRCT. Another group recently published that lymphatic impairment worsened the atherosclerosis plaque formation in atherogenic LDLR^-/-^/ApoB^100/100^ mice crossed with transgenic mice baring lymphatic localized insufficiency, without, however, affecting the RCT rate [45]. In addition to being a key element in promoting the cholesterol efflux from the atherosclerotic lesion, the lymphatic network is thought to play a crucial role in the transport of immune cells involved in the inflammatory response driving plaque progression [47].

## Lymph composition in lymphatic function

One of the main role of the lymphatic system is to preserve fluid homeostasis within the body [15] by absorbing lymph from the peripheral tissue and bringing it back to the bloodstream. Lymph is thus rich in lipoproteins such as HDL, immune cells, electrolytes, nutrients and antibodies. Lymph composition influences flow rate, and is believed to modulate lymphatic function *per se*.

Since the early 1970s, it was well known that cardiac lymph originates from the interstitial fluid, and in 1972, Ulall *et al.* determined the flow characteristics and composition of normal heart lymph that would serve as an essential baseline for future observations [48]. The average lymph chloride and sodium concentrations were higher, whereas the potassium concentration was lower, when compared with blood. Furthermore, all the protein fractions were present in cardiac lymph, but in different concentrations and proportions, as the albumin/globulin ratio was higher in cardiac lymph. The cardiac lymph showed a significantly higher mean lactate level when compared with the coronary sinus blood samples, and the cardiac lymph pH was situated at around 8.0 or higher [48]. By 1983, Sloop *et al.* further characterized the chemical composition and physical appearance of peripheral lymph HDL that was markedly different from that of plasma HDL, especially in cholesterol-fed dogs [49,50]. Lymph HDL had higher cholesterol to protein ratio and markedly increased free cholesterol content when compared with plasma HDL. The phospholipid content of lymph HDL was higher than that of plasma HDL, while the protein content was lower.

Although peripheral lymph lipoproteins have been characterized in animals, there is scarce information about their composition, and close to none about their ultrastructure, in normal humans. There are however, some studies that have started to emerge, analyzing human lymph [51]. Several studies have confirmed that lymph composition is different than that of plasma or serum. Back in 2000, Nanjee *et al.* elucidated the fact that the concentration of small prebeta HDLs in human tissue fluids is determined only in part by their transfer across lymphatic capillary endothelium from plasma. They showed that local production, by remodeling of spheroidal HDLs in tissue fluids, is just as important [52]. Continuing down this path, in 2001, Nanjee *et al.* made observations regarding the composition, as well as the ultrastructure of different subclasses of normal human peripheral lymph lipoproteins [53]. Apolipoprotein B was found almost exclusively in low density lipoproteins, and more importantly, total cholesterol concentration in lymph HDL was 30% greater than could be explained by the transendothelial transfer of HDL from plasma, which provided direct confirmation that HDL acquires cholesterol in the extravascular compartment [53].

We now know that a broad array of cytokines, proteins, growth factors are contained within lymphatic fluid, which play an important role in metabolism, proliferation, as well as an immunoregulatory role [54]. When comparing with serum concentrations, Zaleska *et al.* concluded the existence of a local autonomous regulatory humoral mechanism in tissues, not reflected in serum, after assessing the fact that local cells contribute to lymph concentration by own production.

## Propelling the lymph down the road: the physiology of the contraction

Lymph production has a significant impact on lymphatic vessels capacity to actively participate to lymph flow [55]. Lymph transport throughout the lymphatic network is regulated by different mechanisms that are either extrinsic or intrinsic to the lymphatic vessels. The relative importance of intrinsic and extrinsic pumping mechanisms varies from a lymphatic bed to another. The extrinsic mechanisms include lymph generation, arteriolar gradient and surrounding muscle contraction (skeletal or smooth muscle). Active transport of lymph is also possible through contraction of collecting lymphatic vessels, considered as intrinsic lymph transport mechanism. Since changes in pressure and flow are both causes and effects of adaptive processes, it becomes crucial to study the adaptation of the lymphatic network. Therefore, a recent study by Dongaonkar *et al.* attested the changes in mesenteric lymphatic muscle mechanical properties and the intracellular Ca^2+^ in response to sustained mesenteric venous hypertension [56]. They showed that following 3 days of mesenteric venous hypertension, the adaptive response of postnodal mesenteric lymphatic vessels resulted in weaker pumps with decreased cytosolic Ca^2+^ concentration. So when it comes to the lymphatics role in mRCT, it is no surprise that further understanding of the physiological characteristics of lymphatic vessel pumping and general dynamics is required.

Although LECs lining the lumen of the vessel are important modulators of lymphatic contraction, lymphatic SMC (LSMC) are described as the active components, generating both force and rhythmicity responsible to lymph flow. LSMCs wrap lymphangions in a disorderly mesh-like structure [57]. Accordingly, lymphatic valves function (open–close transitions) is passive and responds to differential pressure between pre- and post-valve lymphagions [58]. Conversely, lymphangion contraction is an active process, requiring the generation of force by the smooth muscle cells. Such process mainly depends on myocyte intracellular Ca^2+^ levels. As described in the following section, most mechanisms triggering or modulating vessel phasic or tonic contraction are influencing LSMCs cytoplasmic Ca^2+^ levels. Increase in cytoplasmic Ca^2+^ leads to its complexing with calmodulin, which activates myosin light chain kinase (MLCK). MLCK stimulates generation of force by the myosin/actin interactions and movement. As in blood vessels, Ca^2+^ dependence of the contractile apparatus can also be modulated and has a significant impact on myocyte contraction [59,60]. Although currently understudied, this regulatory pathway is nonetheless quite effective. However, its involvement in the regulation of lymphatic vessels active pumping might be more relevant in pathological conditions.

Lymph flux upstream will increase intraluminal pressure within a lymphangion, producing distension of the vessel wall. Such stretch will trigger a myogenic response from the LSMCs. The ensuing contractions of the smooth muscle cells will then increase intraluminal pressure and propel the lymph through the following valve and lymphangion [61]. Although the specific components integrating vessel wall stretching into contraction and myogenic contraction remains to be clearly established, membrane potential is depolarized in stretched LSMCs [62].

In addition to transluminal pressure, lymphatic contraction is sensitive to lymph flow. Every contraction stroke is associated with a pulsatile fluid movement, a nonlaminar lymph flow due to the presence of valves. The subsequent shear stress stimulates endothelial cells, which modulate LSMCs contraction [63,64]. However, there are several limitations to interpreting the role of shear stress in this process. For example, the impact of shear stress on endothelial activity is generally studied at supraphysiological levels of shear stress. Although shear stress in lymphatic vessels is estimated around 0.6 dyn/cm^2^, endothelial activation reaches a plateau when exposed to shear stress higher than 3 dyn/cm^2^. These experiments are generally carried out in cultured cells, where phenotype alteration might result in a significant modification of the effect observed. Moreover, loss of communication with the underlying smooth muscle could also be responsible for changes in sensitivity to lymph flow. However, mathematical models strongly suggest that differential flow is responsible for higher NO concentration near lymphatic valves [65].

Lymphatic contraction is essentially depending on intrinsic LSMCs properties. However, the myocytes undergo several regulatory influences, including humoral and neural. The most important modulator of LSMCs contraction is the endothelium as endothelium strongly regulates lymphatic smooth muscle electrical excitability and contractility. This control occurs mainly through the generation and release of nitric oxide (NO), although other endothelial derived vasoactive molecules including but not limited to arachidonic acid derivatives (e.g., prostacyclin, thromboxane A2) and endothelin-1 have also been reported to modulate lymphatic contraction dynamics. However, several other candidates in endothelial-dependent modulation of lymphatic contraction have not been explored yet. For example, local ATP release by Pannexin channels could activate nearby purinergic receptors. Similarly, opening of endothelial K^+^ channels such as K_Ca_2.3 or K_Ca_3.1 channels might lead to ‘K^+^ clouds’, activating smooth muscle Kir channels and promoting its relaxation (or a decrease in phasic contractions) via myocyte hyperpolarization. Interestingly, VEGF-C through its binding to VEGFR-3 has also been reported to modulate lymphatic pumping as positive chronotrope and increased lymphagion dilation [66].

Phasic contractions of lymphangions are also spontaneous as they can occur in the absence of stimulation. Autonomous contraction is triggered by LSMCs action potentials (APs). Interestingly, tight electrical coupling between LSMCs allows synchronization of myocytes electrophysiological state and coordinated contraction within individual lymphangions. APs result from the summation of spontaneous transient depolarisations (STDs) occurring in LSMCs and might lead to myocyte contraction [67]. Myocyte stretching results in an increase in STD and AP frequency and the associated contraction. STDs originates from a spontaneous IP_3_ receptor-dependent Ca^2+^ release that activates Ca^2+^-activated Cl^-^ channels (ClcCa) [62]. Molecular identity of ClcCa remains to be established but growing body of evidence suggests that vascular ClcCa might be encoded by TMEM16A. As of now there is no information available on the impact of the loss of TMEM16A expression on lymph propelling, but deleterious effects on lymphatic function can be hypothesized. Likewise, pathological conditions associated with dysfunctional lymphatic vessel contraction could involve alteration of TMEM16A functional expression. On the other hand, Bestrophins can encode Ca^2+^-activated Cl^-^ channels and might also represent lymphatic ClCa, stressing out the requirement of further investigation. Despite its unresolved identity, repolarizing currents by Clca will increase open probability of L-type Ca^2+^ channels, increasing Ca^2+^ influx and leading to contraction. Similarly to blood vessels, the main voltage-dependent Ca^2+^ channel involved appears to be Cav1.2 but a contribution of T-type Ca^2+^ channels to lymphatic pacemaking capacity have also been reported [68]. Several other ion channels recently found to be important in blood vessels (e.g., two-pores K^+^, KCNQ channels) have not been studied in lymphatic vessels. Their potential absence in physiological condition does not warrant unequivocal exclusion of pathological involvement.

The mechanisms responsible for the spontaneous ER Ca^2+^-store release leading to STDs remain to be established. A Ca^2+^-induced Ca^2+^ release coupling might be involved, where small Ca^2+^ entry would favor IP_3_R opening and Ca^2+^ release [69]. Alternatively, endothelial Ca^2+^ influx pathways in lymphatic vessels are currently unknown other than the voltage-dependent Ca^2+^ channels. Recent exciting work on local Ca^2+^ dynamics in blood vasculature brings enthusing hypotheses. Indeed, local Ca^2+^ entry through TRP channels such as TRPV4 [70] or TRPA1 [71] could lead to local Ca^2+^ increase, with little effect on global Ca^2+^. Moreover, Ca^2+^ microdomains appears to be tightly regulated by multiprotein complexes [71,72]. Local Ca^2+^ entry through voltage-dependent Ca^2+^ channels (like Ca^2+^ sparklets) might also be involved like in blood vessels.

Despite the fact that each lymphangion contraction dynamic is independent (they can be autonomous), there is strong evidence that interlymphangions electrical communication allows a synchronization of the vessel contraction and improves stroke efficiency [57]. Intercellular communication is relying on expression of gap junction channels, complexes of connexins, well characterized in lymphatic and blood vessels [73,74]. Interestingly, electron microscopy studies showed the presence of myoendothelial projections (MEPs) through the basal lamina [75]. These MEPs are in fact privileged sites of communication between endothelium and myocytes, where clustering of specialized protein complexes allow specific signaling and microdomains. Direct electrical coupling does not seem to occur [76] but is not necessary to endothelial-smooth muscle communication as diffusible molecules can be involved, as ‘K^+^ clouds’ or even NO production in these juxtaposed structures.

## Conclusion & future perspective

Tremendous progresses have been made toward understanding the mechanisms bridging lymphatic biology to chronic inflammatory disease such as atherosclerosis. Development of genetic mouse models, surgical methods and functional tools has contributed to the exponential advances made in the field in the last decades. Notwithstanding of the significant advances, it is clear from the research highlighted in this article that there is still much to learn about the role of the lymphatic network in coronary heart disease. In the near future, new therapies targeting the functional link between lymphatic dysfunction and atherosclerosis are predicted to emerge with continuously promising results, bringing forward novel perspectives for the treatment of atherosclerosis.

**Executive summary**

**Background**

Tools to study the venous and arterial circulation have been developed at a greater pace, while exploring the lymphatic network had its load of difficulties.
Tremendous progress in lymphatic biology has been made since the turn of the present century: new genetic mouse models and imaging tools, developed for both animals and humans.
This article highlights how progress has contributed to unraveling the role of the lymphatic system in different pathophysiologic conditions, such as in atherosclerosis.

**Historical perspective**

While practicing vivisection on a dog, Gasparo Aselli and a group of colleagues depicted the ‘vessels containing white blood’ described by Hippocrates in 400 BC.

**General anatomy & functions of the lymphatic vessels**

The lymphatic network is an essential component of the immune system, and is required for the maintenance of fluid homeostasis within the body.
Lymphatic vessels absorb lymph through blind-ended lymphatic capillaries, which then converges into larger precollecting and subsequently collecting lymphatic vessels, responsible of maintaining lymph flow through contraction of units called lymphangions.

**Lymphatic morphology in atherosclerosis**

Lymphatic vessels arepresent in the aortic wall, mostly in the adventitia.

**Lymphatic function in atherosclerosis**

Recently, a functional and quantitative study has reported the prerequisite role of the lymphatic system in the removal of cholesterol from the artery wall, putting front stage the importance of the lymphatic network in mRCT.

**Lymph composition in lymphatic function**

Lymph is rich in lipoproteins such as HDL, immune cells, electrolytes, nutrients and antibodies.
Lymph composition influences flow rate and is believed to modulate lymphatic function *per se*.

**Propelling the lymph down the road: the physiology of the contraction**

Transport throughout the lymphatic network is regulated by different mechanisms that are both intrinsic and extrinsic to lymphatic vessels.
Lymphangion contraction is an active process, requiring the generation of force by smooth muscle cells and modulated by the endothelium.

**Conclusion & future perspective**

Tremendous progresses have been made toward understanding the mechanisms bridging lymphatic biology, to chronic inflammatory disease such as atherosclerosis.
Translational studies need to further evolve, in order to soon be able to use lymphatic dysfunction as a clinical marker essential for cardiovascular diseases prevention.
